# A Treatise on Gout

**Published:** 1890-03

**Authors:** 


### Reviews of Books.

---

*A Treatise on Gout.* By Sir Dyce Duckworth, M.D. Edin., Physician to, and Lecturer on Clinical Medicine in, St. Bartholomew's Hospital, &c., &c. With Frontispiece and Illustrations. London: Charles Griffin & Co. 1889.

After having done more than observe the advice which Horace gives to authors intending to publish, Sir Dyce Duckworth has at length conferred upon the profession the welcome boon of issuing his long-contemplated work on gout. Amid all the high-sounding phrases and the straining after scientific precision with which modern medicine abounds, it seems suggestive of Arcadian simplicity to read a book by an author of Sir D. Duckworth's eminence, in which are upheld the importance of treatment, the validity of the doctrine of diatheses, and superiority of English theories and practice to those of Germany or the United States. Explicitly professing himself not to be a specialist, and seeing gout therefore in its full relation to other diseases, Sir Dyce justly remarks that many years' experience of hospital and private practice in London has afforded him in this, the largest field for observation of gout in the world, such opportunities of acquiring an experience, which it is only right should be utilised for the benefit of the profession at large. This he has done with authority and success. We shall not subject our author to a detailed review, or even give an analysis of his work; everyone practising medicine must possess the book, which will take the place hitherto held by Sir A. Garrod's *Treatise*. We will content ourselves with one or two remarks only. On the subject of the pathogeny of gout, Sir Dyce is now much less dogmatic upon its owning a neurotic cause than some years ago he was in his article in *Brain*, when he considered that the portion of the nervous system implicated is situate in some part of the medulla oblongata; assuming now the existence of a basic arthritic diathesis, gout, as well as rheumatism, he considers to be a neuro-humoral disease. Could not as much be said of all other dyscrasiæ? The section on treatment is full and most helpful, and will always be referred to with much advantage by the busy practitioner. The lines of familiar English modes of treatment are mainly advised, no allusion being made to the recommendations to treat gout on opposite principles of dietetics which have been lately advocated on the Continent. In support of his adherence to well-tried remedies and *régime*, our author quotes from *The Taming of the Shrew*, "Old fashions please me best; I am not so nice to change true rules for *new* inventions"; which, with the utmost deference for our learned and accomplished author, Bianca does not precisely say. The book is adorned with a few excellent illustrations, but if amongst our lively expression of gratitude to Sir Dyce we may find a fault, it would be to remark that to the various representations of microscopical sections there is nothing given by way of indicating the scale upon which they are drawn. This is but a needle in a bottle of hay, and will hardly be discovered; but your critic is nothing if not captious.

